# Recent nanotechnology advancements to treat multidrug-resistance pancreatic cancer: Pre-clinical and clinical overview

**DOI:** 10.3389/fphar.2022.933457

**Published:** 2022-08-24

**Authors:** Abdullah K. Alshememry, Nasser B. Alsaleh, Nora Alkhudair, Rami Alzhrani, Aws Alshamsan

**Affiliations:** ^1^ Department of Pharmaceutics, College of Pharmacy, King Saud University, Riyadh, Saudi Arabia; ^2^ Nanobiotechnology Unit, College of Pharmacy, King Saud University, Riyadh, Saudi Arabia; ^3^ Department of Pharmacology and Toxicology, College of Pharmacy, King Saud University, Riyadh, Saudi Arabia; ^4^ Department of Clinical Pharmacy, College of Pharmacy, King Saud University, Riyadh, Saudi Arabia; ^5^ Department of Pharmaceutics and Pharmaceutical Technology, College of Pharmacy, Taif University, Taif, Saudi Arabia

**Keywords:** multidrug resistance, drug delivery, paclitaxel, tumor microenvironment, pancreatic cancer, non-clinical studies, clinical studies

## Abstract

Pancreatic cancer (PC) remains one of the most lethal and incurable forms of cancer and has a poor prognosis. One of the significant therapeutic challenges in PC is multidrug resistance (MDR), a phenomenon in which cancer cells develop resistance toward administered therapy. Development of novel therapeutic platforms that could overcome MDR in PC is crucial for improving therapeutic outcomes. Nanotechnology is emerging as a promising tool to enhance drug efficacy and minimize off-target responses via passive and/or active targeting mechanisms. Over the past decade, tremendous efforts have been made to utilize nanocarriers capable of targeting PC cells while minimizing off-target effects. In this review article, we first give an overview of PC and the major molecular mechanisms of MDR, and then we discuss recent advancements in the development of nanocarriers used to overcome PC drug resistance. In doing so, we explore the developmental stages of this research in both pre-clinical and clinical settings. Lastly, we discuss current challenges and gaps in the literature as well as potential future directions in the field.

## Introduction

Pancreatic cancer (PC) accounts for 2.5% of all cancer cases worldwide, making it the fourth leading cause of cancer mortality (Siegel et al., 2019). An estimated 495,773 patients were diagnosed with PC in 2021 (Cancer.Net, 2022), with approximately 50% of the cases were diagnosed with metastatic disease (Bray et al., 2018). The location of the tumor within the pancreas can influence the symptoms and clinical presentation (Ryan et al., 2014; Ducreux et al., 2015; Tempero et al., 2019). Almost 65% of PC tumors develop in the head and the neck of the pancreas, and patients commonly present with jaundice and abdominal pain due to bile obstruction (Ducreux et al., 2015; Tempero et al., 2019). In some cases, PC can either be located in the pancreas body (15%), and the tail (10%), or can be a multifocal tumor (2%). Furthermore, patients can present with other non-specific symptoms, including late-onset type II diabetes, abdominal pain, weight loss, and steatorrhea (Gheorghe et al., 2020).

Pathologically, PC originates from two types of cells: exocrine or endocrine cells (Bardeesy and DePinho, 2002; Ghaneh et al., 2007; Corbo et al., 2012; Aguirre and Collisson, 2017). Over 95% of all PC types are of exocrine origin, where the majority of these cases are pancreatic adenocarcinomas (PDAC) (Cowgill and Muscarella, 2003; Li and Jiao, 2003). Less common cells are those of acinar and cystadenocarcinoma origin (Table 1). The endocrine tumors include neuroendocrine tumors (PNET), which account for 5% of all PC cases (Hackeng et al., 2016). PNETs grow slowly and are less aggressive than the PDAC (Bardeesy and DePinho, 2002; Ghaneh et al., 2007). PDAC is a complex disease with poorly differentiated histological features. The high intertumoral heterogeneity, genomic instability, and stromal desmoplasia formation cause significant challenges in the early diagnosis and treatment of PDAC (Ghaneh et al., 2007; Corbo et al., 2012; Aguirre and Collisson, 2017). Pancreatic intraepithelial neoplasia (PanIN) is the most common early non-invasive precursor lesion of PDAC, in addition to mucinous cystic neoplasm and intraductal papillary mucinous neoplasm. The precursor’s progression from low-grade to high-grade is due to genetic and epigenetic alterations that lead to the formation of invasive PDAC. When PDAC lesions form, the genetic mutations will continue to progress beyond primary mutations (Ghaneh et al., 2007; Rishi et al., 2015; Hackeng et al., 2016; Dreyer et al., 2017).

**TABLE 1:** Pancreatic cancer pathological types. Pancreatic cancer pathologically originates from exocrine or endocrine cells. The prevalence and common mutations are different depending on the tumor type and cell origin. Invasive ductal adenocarcinoma is a common type of PC in cells with an exocrine origin.

Type	Category	Prevalence	Mutations
Invasive ductal adenocarcinoma	Exocrine	95%	KRAS, P16/CDKN2A, TP53, SMAD4
Acinar cell carcinoma	Exocrine	1–2%	APC/β-catenin
Serous Cystadenocarcinoma	Exocrine	3%	VHL
Neuroendocrine (PENT)	Endocrine	5%	MEN1

KRAS: Kirsten rat sarcoma viral oncogene homolog; CDKN2A: Cyclin-dependent kinase inhibitor 2A; TP53: Tumor protein p53; SMAD: an acronym from the fusion of *Caenorhabditis elegans Sma* genes and the *Drosophila Mad*; APC: Adenomatous polyposis coli; VHL: Von Hippel-Lindau; MEN1: Multiple endocrine neoplasia type 1.

Drug resistance, both intrinsic (innate) and acquired (in response to drug therapy), is a key contributing factor to the poor prognosis of PC (Binenbaum et al., 2015). The survival rate of PC has remained almost unchanged for several decades, and it is considered among the lowest, with a 5-year survival rate for a maximum of 9% of cases for all stages combined (Rawla et al., 2019; Siegel et al., 2019). Such a low rate of survival is attributed to two major factors: 1) late diagnosis of typically advanced/metastasized and unresectable PC due to a lack of early diagnostic biomarkers; and 2) lack of effective therapeutic intervention (Siegel et al., 2019). There are fundamental reasons why pancreatic tumors are difficult to treat in PC. Firstly, pancreatic tumors, such as PDAC, are solid tumors comprised of a dense stromal environment of cancerous cells, non-cancerous cells such as fibroblasts, and a dense extracellular matrix. The density of these arrangements impedes drug permeation. Additionally, if a drug can permeate the cancerous stroma, the drug molecules are typically unable to differentiate between cancerous and non-cancerous cells due to the dense tumor stroma (Heidemann and Kirschner, 1978; Sofuni et al., 2005). An important determinant of peritoneal metastasis is the anatomical position of the primary tumor (Baretti et al., 2019). In some cases, cells directly attach to and invade organs and tissues in the peritoneal cavity (Avula et al., 2020) or result in intraperitoneal metastases via blood vessels or lymphatic absorption through the hematogenous route (Ge et al., 2017). In most cases of PC, metastasis has already occurred by the time of diagnosis. Furthermore, even when surgical intervention is applicable, following chemoradiotherapy, rapid relapse is often seen due to the presence of pancreatic satellite cells that promote carcinogenesis (Hosoki, 1983; Sofuni et al., 2005; Zhi et al., 2014; Binenbaum et al., 2015).

The stromal desmoplastic reactions induced by the pancreatic stellate cells (PSC), when activated by growth factors, lead to the secretion of collagen, hyaluronic acid, and other components of the extracellular matrix. Thus, PSCs induce stromal fibrosis, reduce cellular vascularity, and induce hypoxia. The stromal barrier created by PSC elevates the interstitial fluid pressure and compresses the blood vessels, preventing passive transportation of chemotherapeutic agents and eventually leading to treatment failure (Erkan et al., 2012; Hackeng et al., 2016). Efficacy of Trans-arterial chemoembolization (TACE) with TACE + radiofrequency ablation (RFA) and/or ^125^I radioactive seed implantation for unresectable pancreatic cancer are other approaches that have been studied retrospectively and TACE combined with either ^125^I seed implantation and/or RFA, was shown to have improved treatment response and overall survival rate compared with TACE alone (Das et al., 2019). However, in the same study, the overall survival rates were better in the case of RFA. To summarize, based on these previous data, the sequence of use and the best combination therapy in the case of PC remains to be investigated.

While there are numerous drugs available for the treatment of PC, two nanoparticle formulations, Abraxane^®^ and Onivyde^®^ (irinotecan liposome injection) have been approved as of July 2022 by the US FDA for the treatment of metastasized cancers, in combination with gemcitabine. Abraxane^®^ is a Paclitaxel Albumin-stabilized Nanoparticle Formulation, approved for the treatment of metastasized PC in combination with gemcitabine hydrochloride as a first-line treatment. More recently, the use of nanotechnology for delivering drugs to targeted sites in the body is an inexpensive and effective system for treating diseases and other conditions. Indeed, the use of nanotechnology can revolutionize the treatment of cancer, by enabling diagnostic tools for early detection of the disease (Hu et al., 2021) as well as improving drug delivery. In PC, the delivery and distribution of drugs to the tumor are compromised due to intrinsic physical and biochemical barriers, which result in increased interstitial fluid pressure, vascular compression, and hypoxia. Moreover, therapies based on targeting immune responses including therapeutic vaccines, immune checkpoint inhibition, and CAR-T cell therapy often do not show expected responses due to a highly immunosuppressive tumor microenvironment. These two factors present as a major challenge for developing effective therapies against PC. Nanoparticles have been extensively studied as delivery platforms and adjuvants for cancer and other disease therapies. Knowledge gained through using nanocarrier-based systems in other cancer types, combined with the ability to modulate nanocarriers toward targeting multiple MDR mechanisms simultaneously provides an opportunity to enable improvement in drug delivery and enhancing therapeutic outcomes for PC.

## Mechanisms of resistance in pancreatic cancer

Numerous alterations at the genetic, epigenetic, and protein levels are implicated in PC drug resistance (Binenbaum et al., 2015). Although different therapy regimens exist, the current standard of care regimen therapy for PC remains largely dependent on gemcitabine (GEM), which is considered a gold standard in chemotherapy. GEM is a nucleoside analog, which provides only a modest clinical benefit (Burris et al., 1997). Multiple combination regimens of chemotherapeutics such as GEM with 5-fluorouracil (5-FU), cisplatin or paclitaxel (PTX), or FOLFIRINOX (i.e., fluorouracil, leucovorin, irinotecan, and oxaliplatin alone or alongside targeted therapies [e.g., cetuximab and bevacizumab]) have failed to demonstrate significant clinical benefits (Berlin et al., 2002; Cascinu et al., 2006; Heinemann et al., 2006). Despite the initial response to chemotherapy in the different forms of PC, the rapid development of drug resistance remains a major challenge in the treatment of PC (Binenbaum et al., 2015).

It is now well established that a variety of cancers mediate their aggressiveness and resistance to chemoradiotherapy via modulating key cellular regulatory pathways that control cell proliferation and differentiation, inflammation, and programmed cell death pathways, including apoptosis and autophagy (Xia et al., 2014). PC shows a significant up-regulation of ATP binding cassette (ABC) transporters ABCB4, ABCB11, ABCC1, ABCC3, ABCC5, ABCC10, and ABCG2 at the RNA level in tumors relative to the normal pancreas (Mohelnikova-Duchonova et al., 2013). Additionally, drug efflux pump MDR1/P-gp is highly expressed in PC cells (O'Driscoll et al., 2007), which may play a critical role in the development of resistance to chemotherapeutic agents. Therefore, it is equally important to understand the underlying molecular mechanisms of PC drug resistance for unraveling novel therapeutic interventions with improved efficacy. In addition, somatic mutations in key genes such as many proto-oncogenes (e.g., Ras, Myc, Cdk4) are critical in the initiation and progression of malignant tumors. However, cancer treatment is even more challenging because tumor exposure to therapy, including chemoradiotherapy and targeted therapy, is often associated with further mutations and the development of compensatory mechanisms that render cancer refractoriness to therapy and increased aggressiveness and metastasis (Binenbaum et al., 2015). Therefore, understanding the molecular mechanisms of drug resistance is crucial in order to intervene and eventually win the battle against cancer.

The main mutations of PDAC include KRAS, CDKN2A, TP53, and SMAD4 (Hackeng et al., 2016). Over 90% of PC is associated with KRAS mutations, most commonly KRAS^G12D^, during both the initial stage (i.e., precursor lesions that develop into invasive pancreatic ductal adenocarcinoma, also known as pancreatic intraepithelial neoplasia) and progression stage (di Magliano and Logsdon, 2013). There have been efforts to discover new PDAC targeting agents. A phase 1 clinical trial (NCT04117087) used a long peptide vaccine combined with Nivolumab and Ipilimumab for resected MMR-p colorectal and pancreatic cancer patients. Interestingly, Govindan *et al.* discovered that AMG 510 is a novel small molecule that can bind specifically and irreversibly in KRAS^G12C^ (Govindan et al., 2019; Alzhrani et al., 2021). KRAS^G12C^ is a mutation that is predominantly found in non-small lung cancer. However, the use of AMG 510 is limited in PDAC because KRAS^G12C^ mutation only accounts for 2%. These findings will motivate the scientific community to develop new drugs that can target inactive KRAS^G12D^ and KRAS^G12V^, which account for 80% of PDAC (Govindan et al., 2019; Alzhrani et al., 2021).

**FIGURE 1:**
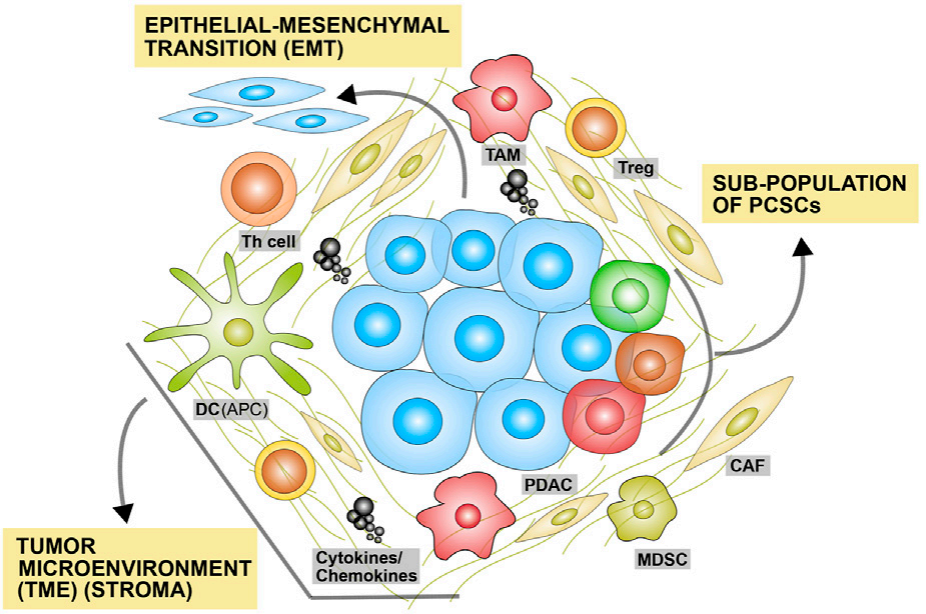
Major molecular contexts underlying drug resistance in pancreatic cancer. This figure illustrates the three major molecular contexts underlying drug resistance in pancreatic cancer. These include: 1) the process of epithelial-mesenchymal transition (EMT) in which cancer cells lose their epithelial phenotype (such as cell-to-cell contact) to gain more aggressive and metastatic mesenchymal phenotypes; 2) expansion of subpopulation of pancreatic cancer stem cells (PCSCs); and 3) dynamic tumor microenvironment (TME). The tumor stroma surrounding cancer cells and PCSCs consist non-cellular components including extracellular matrix (ECM) and multiple cell types including CAF, MDSC, Th-cells, Treg, TAM and DC. Cytokines released from the different cell types help maintaining the TME in dynamic state that supports tumor growth and metastasis. Th-cells exist in TME but are suppressed. DCs, which are key in the processing and presentation of cancer neoantigens, exist in TME but they are suppressed as well. EMT: epithelial-mesenchymal transition; PCSC: pancreatic cancer stem cell; TME: tumor microenvironment; CAF: Cancer-associated fibroblast; MDSC: myeloid-derived suppressor cell; Th-cells: Helper T cells; Treg: Regulatory T-cell; TAM: tumor-associated macrophage; DC: Dendritic cell.

It is worth noting that tremendous efforts over the past years have revealed numerous molecular components and intricate signaling networks that are deregulated in PC, contributing to chemoresistance. These include, but are not limited, drug transporters (e.g., hENT and hCNT), intracellular enzymes (e.g., deoxycytidine kinase), dCK, which is critical for GEM bioactivation, DNA repair mechanisms (e.g., excision repair cross-complementation 1), ERCC1, antioxidant response (e.g., Nrf and HSPs), signaling pathways that regulate cell-cycle and programmed cell death (e.g., Nuclear Factor κB [NFκB], MAPK, PI3K/Akt and p53), epigenetic components (e.g., histone deacetylase), HDAC and more recently, noncoding RNAs (ncRNAs) [e.g., microRNAs (miRNAs), long noncoding RNAs (lncRNAs) and circular RNAs (circRNAs)] (Binenbaum et al., 2015; Xie et al., 2020a; Lin et al., 2020; Pandya et al., 2020). More recently, differential upregulation and functional role of the transmembrane mucin MUC4 in PC is an attractive target for immunotherapy and MUC4β encapsulation in polyanhydride nanoparticles has been shown to provide long-term protection against rapid phagocytic and proteolytic clearance in circulation. Stable MUC4β release from these nanoparticles and its immunogenic capacity has recently been demonstrated by Liu and colleagues in mice models (Liu et al., 2021). Discussing the specific molecular mechanism is beyond the scope of this review and is discussed in detail elsewhere, but here we give an overall overview and describe three key themes involved in PC drug resistance. These are epithelial-mesenchymal transition (EMT), expansion of pancreatic cancer stem cells (PCSCs), and dynamic state of the tumor microenvironment (TME), as illustrated in (Figure 1) (Binenbaum et al., 2015; Zeng et al., 2019).

Epithelial-mesenchymal transition (EMT) is a dynamic process that involves the transitioning of differentiated epithelial cells into the mesenchymal cell phenotype. During this transformation, cells lose key features of epithelial cells such as intercellular junctions while simultaneously undergoing cytoskeletal rearrangement of the mesenchymal cell phenotype (Nieto et al., 2016). EMT is particularly critical during cancer progression and the generation of stem-like cells with a high capacity for metastasis (Nieto et al., 2016). Notably, previous literature has demonstrated the key role of EMT in conferring drug resistance against chemoradiotherapy and targeted therapy in a variety of solid tumors, including PC (Shah et al., 2007; Wang et al., 2009; Singh and Settleman, 2010; Nieto et al., 2016). For instance, chemoresistance to GEM in PC cells has been associated with increased expression of mesenchymal markers such as vimentin and ZEB1, in contrast to GEM-sensitive cells with a high E-cadherin expression: a common epithelial phenotype maker (Li et al., 2009). Similar findings have been found with other chemotherapeutics, such as 5-FU and cisplatin (Arumugam et al., 2009). Although the underlying molecular mechanisms of EMT-induced chemoresistance are not fully understood, accumulating evidence suggests the involvement of multiple signaling pathways, including NFκB, TGFβ, and Notch pathways (Ellenrieder et al., 2001; Min et al., 2008; Wang et al., 2009). Another key molecular regulator of EMT in PC cells is miRNAs: small non-coding RNAs that regulate gene expression (Yonemori et al., 2017). A multitude of miRNAs has been shown to be implicated in regulating major cellular signaling pathways involved in the development of PC (Yonemori et al., 2017). For instance, a low expression of miR-200 was found in the GEM-resistant but not in the GEM-sensitive PC cell lines (Li et al., 2009). Together, these findings indicate the critical role of EMT in the development of PC drug resistance in transformed cells.

Previous evidence has demonstrated that cancers are heterogenous in nature, with subpopulations of cells of varying phenotypes. Cancer stem cells (CSCs) represent a small subpopulation of tumor cells with a high capacity for self-renewing, differentiation, and tumor progression (Yu et al., 2012). Indeed, CSCs display a high capacity for tumorigenesis when transplanted into a host compared to other tumor subpopulations (Yu et al., 2012). Several cellular surface markers have been identified to be expressed by CSCs in various cancers, including CD44, CD24, CD133, aldehyde dehydrogenase1 (ALDH1), and epithelial-specific antigen (ESA) (Yu et al., 2012). In PC, previous work has demonstrated that CD44, CD24, and ESA-expressing PCSCs, represented only by a small percentage of the cancer cell population (∼1%), were associated with high tumorigenic potential, and it is likely that PCSCs at least partially mediate chemoradiotherapy-induced drug resistance, as evident in PCSC enrichment in response to chemoradiotherapy treatment (Du et al., 2011). Similar findings were also found in another report, demonstrating that within a pancreatic tumor, a subset of undifferentiated, stem-like cells expressing the surface marker CD133, was associated with high tumorigenic potential and chemoresistance (Hermann et al., 2007). Although there is an overlap between the two subpopulations of PCSCs in the two reports (CD44/CD24/ESA-vs. CD133-expressing PCSCs), they were not identical (Hermann et al., 2007). Additionally, another subset of cells with exclusive migratory properties expressing CD133 and CXCR4 were identified at the invasive front of pancreatic tumors. It was found that without these CD133^+^/CXCR4^+^ cells, metastasis was almost completely abrogated (Hermann et al., 2007). Together, these findings indicate the existence of different subsets of PCSCs within pancreatic tumors with a high capacity for tumorigenesis and drug resistance. Such findings demonstrate the heterogenic nature of cancers which has been long disregarded in cancer research. It is worth noting that EMT transformed cells can share cellular and molecular features of CSCs, which could explain an increased tumorigenic profile of EMT transformed cells as well as drug resistance. Nonetheless, these cells, unlike PCSCs, were sensitive to conventional chemotherapy. Furthermore, it is now widely accepted that subpopulations of CSCs exist in a dynamic state (quiescent vs. slow-vs. rapid-cycling CSCs), which is constantly influenced by signals and cues from their surroundings (the tumor microenvironment (TME). With their large plasticity and ability to switch to a quiescent state, CSCs can resist chemoradiotherapy (Batlle and Clevers, 2017).

The tumor microenvironment (TME) consists of cellular and non-cellular components (extracellular matrix (ECM)) and plays a crucial role in drug resistance. Indeed, one major obstacle in the treatment of PC is the dense fibrotic stroma, also known as desmoplastic stroma, surrounding tumor cells that act as a physical barrier. As such, they prevent drugs from infiltrating tumor core cells (Dauer et al., 2017). Thus, the dense fibrotic stroma is considered a histopathological hallmark of PC. It is worth noting that TME is composed of several types of cells, including fibroblasts, pancreatic stellate cells (myofibroblast-like cells), and a variety of immune cells and despite the considerable efforts made to target a multitude of cellular and noncellular components within TME, yet it was associated with limited clinical success (Ho et al., 2020). For instance, within the stromal cells, cancer-associated fibroblasts (CAFs), which are the major source of ECM, are associated with immunosuppressive properties (Kraman et al., 2010). Depletion of CAFs was associated with enhanced efficacy of immune checkpoint inhibitors (ICIs) (Feig et al., 2013). In addition to CAFs, other cell types within the TME including regulatory T-cells (Treg), myeloid-derived suppressor cell (MDSC), and tumor-associated macrophage (TAM) also possess immunosuppressive properties (Ho et al., 2020). Cancer immunotherapy has revolutionized the field over the past couple of decades. Notable success has been made against multiple solid cancers, particularly those with high tumor mutational burden (TMB) within the tumor genome, such as melanoma and lung cancer (Goodman et al., 2017; Yousefi et al., 2017). This success has not been evident in PC (Schizas et al., 2020), which has largely been attributed to the non-immunogenic nature of most types of PC. In other words, most PC types are not readily recognized by the immune cells due to low TMB and thus a low number of neoantigens, a tumor-associated antigen, and subsequent presences of TILs (Bailey et al., 2016; Danilova et al., 2019). Additionally, as mentioned earlier, PC is typically contained in a highly dense fibrotic stroma consisting of multiple cell types with immunosuppressive properties (Ho et al., 2020; Schizas et al., 2020). Unsurprisingly, together, these two factors make cancer immunotherapy less effective in PC. Utilizing the properties of TME for controlling local therapeutic delivery is an area of active research (Alshememry et al., 2017).

## Nanotechnology for the treatment of pancreatic cancer

Nanomaterials and nanoparticles (NPs) are extremely small (1–100 nm) in size and hence can directly interact with biological molecules (Hosein et al., 2013). Engineered NPs are an excellent tool for drug delivery due to their unique structural properties which include a large surface-to-mass ratio, capacity to be modulated to bind different cellular targets, and ability to carry different cargo including proteins, nucleotides, and drugs. Advances in the field of nanotechnology have created tremendous prospects for improving therapeutic drug delivery (Schroeder et al., 2011; Melancon et al., 2012; Prabhu and Patravale, 2012). Currently, the use of nanotechnology in drug delivery typically involves a combination of nanomaterials and a drug of interest, and a significant number of nanoplatforms are being employed and are under testing in the different phases of clinical trials (Ogawa and Miura, 2014; Rebelo et al., 2017). These combinations utilize different types of nanomaterials such as polymeric NPs, liposomes, amphiphilic polymer NPs, small interfering RNA (siRNA), graft polymers, dendrimers, thermo-responsive polymers, mixed micelles, ultrasound-responsive nano-emulsions, carbon nanotubes, quantum dots and inorganic NPs (magnetic-hybrid NPs, and gold NPs) (Manzur et al., 2017; Rebelo et al., 2017; Sielaff and Mousa, 2018).

Nanotechnology has been largely utilized in cancer research to improve the delivery of drugs to the tumor site exploiting the leaky vasculature of the tumor via passive or enhanced permeability and retention (EPR) effect. However, a major challenge in PC is its hypovascularization, and hence, other strategies must be utilized, such as using targeted (active) delivery (Xie et al., 2020b). As a result of this insufficient vascularization, ineffective distribution of drugs may account for much of the chemotherapy resistance seen in PC treatment. The use of nanotherapy may bypass the inefficient vascularization issues by delivering chemotherapeutic drugs directly to the pancreatic tissue, for example, by targeting stromal hedgehog receptors rather than relying on blood flow, a methodology that provides a tunable distribution of chemotherapeutic agents throughout pancreatic tumor tissue, thereby increasing drug solubility, half-life, and stability (Jiang et al., 2020). Moreover, NPs delivering chemotherapeutic drugs can bypass multidrug-resistant (MDR) efflux pumps present on the surface of most tumor cells (Lobato-Mendizabal and Ruiz-Arguelles, 1990; Blanco et al., 2011; McCarroll et al., 2014). This, among other reasons that will be discussed in later sections, supports the use of nanotechnology in PC treatment.

## Nano-based intervention to overcome MDR-PC in pre-clinical and clinical settings: Key examples

The use of NPs has several advantages, including minimizing MDR and drug-related toxicities. Nanoparticles can target a wide range of physiological and metabolic characteristics of the targeted tissues, thereby increasing biodistribution and bioavailability of drugs and enhancing their plasma half-life and EPR (Parhi et al., 2012; Poon et al., 2015; Gad et al., 2016; Liu et al., 2022). Recently, the potency of the chemotherapeutic agents for the treatment of PC has been improved through the use of RNA interference (RNAi) technologies, including miRNA and siRNA, which selectively suppress the expression of target genes leading to increased drug efficacy and enhancing anti-cancer activity (Tang et al., 2021). Integrating RNAi with NPs can therefore be extremely effective at treating PC (Hiss et al., 2007; Gurunathan et al., 2018). Studies have recently shown that nano-sized exosomes are an efficient RNAi carrier, making them an attractive delivery cargo to cancerous cells (Farran and Nagaraju, 2020) Exosomes have intrinsic advantages over liposomes, as they are less toxic and can be dosed at higher concentrations in the blood to work as molecular cargos which could be used to inhibit oncogenes, activate tumor suppressor genes and modulate immune responses to control tumor cell growth (Oliveira et al., 2021) (Zhao et al., 2021b). As researchers continue to uncover the cellular and molecular basis of PC drug resistance, nanotechnology used for the delivery of drugs can be further engineered to provide effective solutions for mitigating MDR in the treatment of PC (Zhang et al., 2017). Table 2 summarizes key nano-based systems used for the delivery of antitumor drugs to mitigate MDR.

**TABLE 2:** Selected examples of nano-based formulations used to overcome MDR in tumor cells.

Type of nanocarrier	Objective	Cargo	Cell/Animal model	Effects on MDR	Outcomes/results
M1Exo-GEM-DFX (Zhao et al., 2021a)	M1Exo was engineered as a drug carrier to co-delivery DFX and GEM to overcome the chemoresistance of GEM and improve its therapeutic potential	DFX and GEM	PANC-1 cells	Inhibit cell P-glycoprotein expression	M1Exo-GEM-DFX was able to overcome GEM resistance induced by P-glycoprotein expression *in vitro*
s (DGL)_n_@Apt NPs(Chen et al., 2022)	Modulation of PDAC stromal structure and send chemotherapy drugs to the deep tumor vis the use of Aptamer-decorated hypoxia-responsive nanoparticle s (DGL)_n_@Apt	GEM + STAT3 inhibitor (HJC0152)	Pan02 multicellular spheroids (MCSs) cells / Pan02 xenograft mice	Inhibition of the STAT3 pathway	Triggered by hypoxia, the ultra-small dual-loaded DGL NPs exhibited excellent deep-tumor penetration, promoted drugs endocytosis, and autophagy induction
PEG-Gem-cisPt-MSNs (Tarannum et al., 2022)	Development of two versions of mesoporous silica nanoparticles (MSNs), a dual loaded PEG-functionalized NPs, and MSNs containing Sonic Hedgehog (SHh) inhibitor for stroma modulation and improved delivery	GEM + CisPt	HPAF II and Miapaca-2 cells / HPAF II xenograft mice	Inhibition of sonic hedgehog (SHh) signaling pathway	The sequential combination of CyP-MSNs followed by PEG-Gem-cisPt- MSNs led to (i) effective stromal modulation (ii) increased access to secondary PEG-Gem-cisPt-MSNs at the tumor site (iii) enhanced therapeutic performance in HPAF II xenograft mice
TPMILs (Obaid et al., 2022)	Development of cetuximab (anti-EGFR mAb) targeted photoactivable multi-inhibitor liposomes (TPMILs) co-loaded with lapidated benzoporphyrin derivative (BPD-PC) photosensitizer and irinotecan to remediate desmoplasia, a major contributor to chemoresistance	(BPD-PC) + irinotecan	MIA PaCa-2 + PCAF tumor model	Reduction in stromal collagen density and collagen fiber alignment	Synchronized chemotherapeutic and a photodynamic insult to PDAC tissue was achieved with doubled overall survival
HSA NPs(Guo et al., 2018)	Enhancing the antitumor effect of GEM by the encapsulation into HSA-NPs to overcome GEM resistance in GEM-resistant PC induced by low hENT1 gene expression	GEM	BxPC-3 and SW1990 cells/patient-derived xenograft BALB/c-nu/nu mice model	Inhibit cell proliferation, arrest cell cycle, and trigger apoptosis	GEM-loaded HSA-NPs was able to overcome GEM-resistance induced by low hENT1 expression *in vitro* and *in vivo*
HSA NPs(Han et al., 2017)	Development of tumor microenvironment targeting HSA-GEM/IR780 complex with the redox-responsive release of GEM using GFLG cleavable peptide	GEM + IR780 (NIR dye)	BxPC-3 cells	Induction of apoptosis and Inhibition of cells proliferation	The developed theranostic nanoplatform showed high tissue accumulation and retention with: (i) targeted intracellular drug release, (ii) enhanced tumor inhibition activity (iii) insignificant side effects
Pheophorbide-a conjugated albumin NPs(Yu et al., 2017)	Inhibit PC with lymphatic metastases by the combination of chemotherapy with photodynamic therapy (PDT)	GEM	(BxPC-3-LN7) cells	Increase in drug accumulation in primary tumors as well as metastatic lymph nodes	Developed triple functional system efficiently controlled the release of GEM from the modified NPs and possessed imaging-guided theranostic properties
Nanovector- albumin-bound PTX (MSV/nAb-PTX) (Borsoi et al., 2017)	Enhancing drug transport by increasing caveolin-1 expression (albumin transporter) via combination therapy of MSV/nAb-PTX with GEM	PTX + GEM	L3.6 pl human cells/L3.6 pl—bearing nu/nu nude mice	Increase cellular uptake as a result of GEM-induced high cav-1 expression, which leads to increased transport of nAb-PTX into tumor tissue	GEM enhanced the transport of MSV/nAb-PTX in GEM-resistant pancreatic ductal adenocarcinoma
Chitosan coated solid-lipid NPs (c-SLN) (Thakkar et al., 2018)	To use nano-encapsulated c-SLNs combinations to determine the efficacy of the ACS therapeutic regimen	Aspirin (ASP)+ curcumin (CUR)+free sulforaphane (SFN); ACS	Panc-1 and MIA PaCa-2 cells/LSL-Kras^G12D/+^; Pdx-1^Cre/+^ transgenic mouse model	Increase in drug efficacy	Due to enhanced bioavailability of the combined ACS chemopreventive agents, the dosage for this therapeutic regimen can substantially be reduced, which by virtue reduces any potential serious side effects
SN38 (irinotecan active metabolite) polymeric prodrug-based NPs(Wang et al., 2017)	Development of a nano-based system for effective synergistic therapy to overcome fibroblast-induced drug resistance	GDC-0449 (hedgehog pathway inhibitor)	BxPC-3 cells and MIA PaCa-2 cells/PSCs and BxPC-3—bearing BALB/c nude mice	Increase in drug efficacy by modulating the fibroblast-enriched tumor microenvironment	size-tunable nanoparticles were obtained and controllable loading efficiency, which was directly correlated to the length of the hydrophobic SN38 block
(PLGA-ORM NPs) (Khan et al., 2015)	Providing effective endosomal release to the cytosol	Ormeloxifene	(HPAF-II, AsPC-1, BxPC-3, Panc-1, and MiaPaca)/a BxPC-3 xenograft mice model	Increase in drug efficacy	PLGA-ORM NPs showed substantial antitumor efficacy and effective endosomal release resulted in PC tumor suppression
PLGA-PEG NPs(Elgogary et al., 2016)	Targeting the glutamine metabolism	BPTES	P8, A6L, A32, P198, E3, P215, P10, and JD13D human PC cells/Foxn1nu athymic tumor-bearing nude mice	Increase in drug accumulation	Combination therapy of BPTES-loaded NPs and metformin were shown to be effective in blocking the metabolism of glutamine and glucose
Redox-responsive Apt/CPP-CPTD NPs(He et al., 2018)	Development of sequentially responsive NPs with redox-responsive on-demand drug release and ECM-responsive tumor penetration	Camptothecin prodrug, CPTD	MIA PaCa-2 cells/MIA PaCa-2 orthotopic human PC xenograft bearing nude mice	Enhance cytotoxicity and cellular accumulation	Formulated NPs showed selective accumulation at the tumor site with mild *in vitro* cytotoxicity and good *in vivo* antitumor efficacy
PLGA NPs(Lucero-Acuna et al., 2014)	Enhanced PH-427 delivery to the PC harboring K-ras mutation to overcome the protective stromal layer surrounding the pancreatic tumor	PH-427 (AKT/PDK1 inhibitor)	MiaPaCa-2 harboring K-ras mutation/Orthotopic MiaPaCa-2—bearing mice	Increase in cellular uptake and drug efficacy	PH-427- loaded PLGA NPs resulted in the enhanced therapeutic effect of PH-427 *in vitro* and *in vivo*
PEGylated colloidal gold NPs(Libutti et al., 2018)	Targeting components of the tumor microenvironment responsible for creating high interstitial fluid pressure to improve the delivery of anticancer drugs	TNF and a PTX prodrug	Genetically engineered mice with pancreatic ductal adenocarcinoma	Increase in drug efficacy by tumor IFP reduction	The combination of TNF (targeting tumor vasculature) with PTX (either loaded on the NPs or administered separately) increased the efficacy of the cytotoxic agent
Superparamagnetic iron oxide nanoparticle (SPION) (Khan et al., 2019)	Development of (SPION) loaded with curcumin (SP-CUR), which is known for its anti-inflammatory and antitumor activity, to overcome GEM resistance and enhance its therapeutic potential *in vitro* and *in vivo*	Curcumin + GEM	Panc-1, HPAF, CPSC, and HPSC cells / HPAF-II human PSCs—bearing athymic Nu/Nu mice	Suppression of sonic hedgehog (SHH) signaling pathway and oncogenic CXCR4/CXCL12 signaling axis	Efficient delivery of curcumin was achieved, which also played a role in sensitizing cells to standard GEM therapy
Nanogels (NGs) (Soni et al., 2019)	Development of Cisplatin-loaded mAb-coated NGs for targeted delivery to PCs and the evaluation of antitumor activity in combination with GEM	Cisplatin	T3M4/Luc cells/ T3M4/Luc—bearing Nu-Nu nude mice	Increase in drug efficacy by targeted therapy using an anti-STn antibody (TKH2 mAb)	Enhanced drug delivery, as well as synergistic cytotoxic effect, was observed after sequential exposure of PC cells to GEM followed by CDDP
Fucose-bound liposomes (Yoshida et al., 2012)	Development of liposomal formulation functionalized with l-fucose to target the fucosylated antigens highly expressed on the surface of cancer cells to enhance cisplatin delivery	Cisplatin	BxPC-3, AsPC-1, PK59, and HuCCT1 cell lines/ Subcutaneous model:AsPC-1-bearing mice; Liver metastasis and orthotopic models: BxPC-3-Luc- bearing mice	Increase in cellular uptake and cytotoxicity	Cisplatin-loaded Fucose-bound liposomes were effectively delivered to PC cells and resulted in effective inhibition of tumor growth as well as extending survival in the mouse xenograft models
Au-GO@ZC-DOX stealth nanovesicles (Thapa et al., 2018)	development of pH-triggered stealth nanovesicles for chemophototherapy	DOX	Panc-1 cells and Mia PaCa-2 cells/PANC-1- bearing BALB/c nude mice	Increase in cellular uptake and cytotoxicity	The multi-componential nanovesicle showed effective Macrophage opsonization inhibition, resulting in anti-cancer and anti-migration effects
HA-SMA Micelles (Kesharwani et al., 2015)	Development of functionalized micelles with HA to target the PC overexpressed CD44 receptors to overcome MDR	3, 4-difluorobenzylidene curcumin (CDF)	MiaPaCa-2 and AsPC-1 cells	Inhibition of NF-κB in CD44^+^ cells	The developed nanosystem showed remarkable colloidal stability and sustained drug release and potent anticancer activity
Polymeric Micelles (Xu et al., 2015)	Development (TPGS–GEM) prodrug micelles to protect the drug from enzymatic metabolism	TPGS–GEM (prodrug)	BxPC-3 cells	Enhanced drug efficacy as the micellar formulation protected the drug from enzymatic metabolism	Long circulation half-life of GEM was obtained in addition to enhanced anticancer activity
Ultra-pH-sensitive micelles (UPSM) (Kong et al., 2019)	Development of UPSM improved pH buffer capacity for simultaneous inhibition of lysosomal acidification and enhancement of therapeutic delivery	Triptolide prodrug-	KRAS mutant PANC-1 and MIA PaCa-2/MIA PaCa-2-luc—bearing BALB/C nude mice	Disruption of lysosomal catabolism and growth inhibition of KRAS mutant	The newly developed nanosystem revealed more efficient lysosomal catabolism when compared with conventional lysosomotropic agents. In addition, pH-sensitive UPSM showed significant cytotoxicity when compared to non-pH-sensitive micelles

### Albumin-based nanoparticles in PC

Albumin-based nanoparticles can be utilized as theranostics (i.e., to deliver therapeutic agents and simultaneously used for diagnosis). Albumin is the most abundant plasma protein and known ligand to be associated with a caveolae-mediated endocytosis mechanism. Albumin-based nanoparticles when internalized by the cell *via* caveolae-mediated endocytosis, can overcome the issue of MDR by bypassing and evading ATP-binding cassette (ABC) transporters, which are responsible for the efflux of anticancer drugs and subsequent MDR once released into the cytoplasm (Yuan et al., 2016). Nanoparticle albumin (Nab) is made by mixing human albumin in an aqueous medium under high pressure to form 100–200 nm albumin NPs. These NPs are mixed with chemotherapeutic alkaloids such as PTX (Macarulla et al., 2019). As shown in Table 3, Nab-based delivery systems are the most extensively studied nanocarrier system in the treatment of PC in human clinical trials (Von Hoff et al., 2011; Hosein et al., 2013; Von Hoff et al., 2013; Goldstein et al., 2015; Vogel et al., 2016; Macarulla et al., 2019). A common example of albumin-based nanoparticles is Nab-PTX, an albumin-binding PTX and a microtubule-stabilizing agent known to enhance microtubule polymerization during mitosis leading to cell cycle arrest in the G2/M phases. Based on these properties, Nab-PTX can stop rapid and uncontrollable cell division and help overcome MDR receptor-mediated endocytosis (Demidenko et al., 2008; Guo et al., 2018). Importantly, Nab-PTX has been shown to mitigate MDR in PC (Guo et al., 2018). In 2018, Guo et al. created GEM-resistant pancreatic cells by inducing lower rates of hENT1 expression. These cells were then exposed to free GEM or GEM delivered using human-serum albumin nanoparticles (HSA-NPs). Their results showed that GEM-HSA-NPs was more effective at slowing pancreatic cell proliferation and triggering apoptosis in comparison to free GEM alone, without any increase in toxicity, as shown *in vivo* studies. In phase III clinical trial (MPACT) on previously untreated patients with metastatic PC, a combination of Nab-PTX and GEM increased the median OS of patients receiving nab-PTX and gemcitabine to 8.7 vs. 6.6 months in patients treated with gemcitabine alone (*p* < 0.0001) (van Horssen et al., 2006; Libutti et al., 2010). Furthermore, the combination showed an increase in the cumulative delivery of gemcitabine by 2.8-fold compared to gemcitabine alone (Von Hoff et al., 2011; Von Hoff et al., 2013; Goldstein et al., 2015). Based on the findings of the trial, GEM plus nab-PTX became the first therapy recommended by the National Institute for Health and Care Excellence in the United Kingdom for the management of previously untreated metastatic PC (Von Hoff et al., 2011).

**TABLE 3:** Selected examples of clinical trials applying nano-based formulations in PC treatment.

Study	Testing	Study type	Dosing regimen	Median survival	Main outcomes
Von Hoff et al., 2011 (Von Hoff et al., 2011)	GEM + Nab-PTX	Phase I/II	67 patients 100, 125, and 150 mg/m^2^ nab-paclitaxel plus 1,000 mg/m^2^ gemcitabine on days 1, 8, and 15 every 28 days	12.2 months	Response rate 48%, Overall Survival (OS) 12.2, and 1-year survival rate 48%
NCT00844649					
Hosein et al., 2013 (Hosein et al., 2013)	nab-PTX	Phase II	19 patients were treated with nab-paclitaxel 100 mg/m^2^ on days 1, 8, and 15 of a 28-day cycle	7.3 months	6 months OS 58%, median OS 7.3 months
Von Hoff et al., 2013 (Von Hoff et al., 2013)	GEM + Nab-PTX vs. GEM	Phase III	Group 1: 431 patients given nab-PTX (125 mg/m^2^) followed by GEM (1,000 mg/m^2^) on days 1, 8, and 15 every 4 weeks	Median survival was 8.5 months in GEM + Nab-PTX vs. 6.7 months in the GEM group	Group 1 vs. Group 2: Median survival 8.5 vs. 6.7 months; 1-year survival rate 35vs. 22%; 2-year survival rate 9 vs. 4%; higher risks of peripheral neuropathy and myelosuppression with group 1 compared to group 2
NCT00844649			Group 2: 430 patients given GEM monotherapy (1,000 mg/m^2^) weekly for 7 of 8 weeks (cycle 1) and then on days 1, 8, and 15 every 4 weeks (cycle 2)		
Goldstein et al., 2015 (Goldstein et al., 2015)	GEM + Nab-PTX vs. GEM	Phase III	Patients (n = 861) randomly assigned to receive GEM + Nab-PTX vs. GEM	Median survival was 8.7 months in GEM + Nab-PTX vs. 6.6 months in the GEM group	OS and long-term survival (>3 years) were higher amongst GEM + Nab-PTX compared to the GEM monotherapy group
Update on OS of NCT00844649					
Vogel et al., 2016 (Vogel et al., 2016)	GEM + Nab-PTX vs. GEM	Phase III	Patients randomly assigned to receive GEM + Nab-PTX vs. GEM alone	Median survival was 9.8 months in GEM + Nab-PTX vs. 7.5 months in the GEM group	OS 8% with GEM + Nab-PTX vs. 4% from GEM alone; Overall Response Rate 27 vs. 9% with GEM + Nab-PTX vs. GEM alone respectively
Sub-analysis of NCT00844649					
Macarulla et al., 2019 (Macarulla et al., 2019)	GEM + Nab-PTX vs. GEM	Phase I/II	6 groups inducted in phase I and 2 groups in phase II both using GEM + Nab-PTX at different doses (100 g/m^2^ or 125 mg/m^2^ Nab-PTX + 1,000 mg/m^2^ GEM)	NA	Improvement in overall survival irrespective of the dose of Nab-PTX used
NCT02382263					
Libutti et al., 2010 (Libutti et al., 2010)	CYT-6091 (colloidal gold)	Phase I	3 participants were given 50 mg/m^2^ to 600 mg/m^2^ of rhTNF via the CYT-6091 delivery system	NA	CYT-6091 delivery system led to great tumor tissue concentration of rhTNF compared to normal tissues
NCT00356980					
Stathopoulos et al., 2005 (Stathopoulos et al., 2005)	Lipoplatin	Phase I	Dose starting at 25 mg/m^2^ and was increased by 25–125 mg/m^2^	NA	No significant nephrotoxicity or systemic toxicity noted with this preparation
Greek trial					
Stathopoulos et al., 2005 (Stathopoulos et al., 2005)	Lipoplatin	Phase II	GEM dose 1,000 mg/m^2^ and the lipoplatin dose was escalated from 25 mg/m^2^ to 125 mg/m^2^	3 months	Partial response (>50% tumor reduction) was seen in 2 patients. Stable disease (<25–50% reduction in the tumor) was seen in 14 patients
Greek trial					
Syrigos et al., 2002 (Syrigos et al., 2002)	Docetaxel and liposomal doxorubicin	Phase II	21 patients given docetaxel (80 mg/m^2^), and liposomal doxorubicin (30 mg/m^2^) was administered on day 1, every 3 weeks	10 months	Median survival 10, 1-year survival 33.3%
Greek trial					
Hamaguchi et al., 2007 (Hamaguchi et al., 2007)	NK105 (PTX- polymeric micelles)	Phase I	Initially given 10 mg/m^2^ and successively increased the dose	NA	The size of liver mets reduced by 90% in patients receiving a dose of 150 mg/m^2^ or higher dose

### Metal-based nanoparticles in PC

**FIGURE 2:**
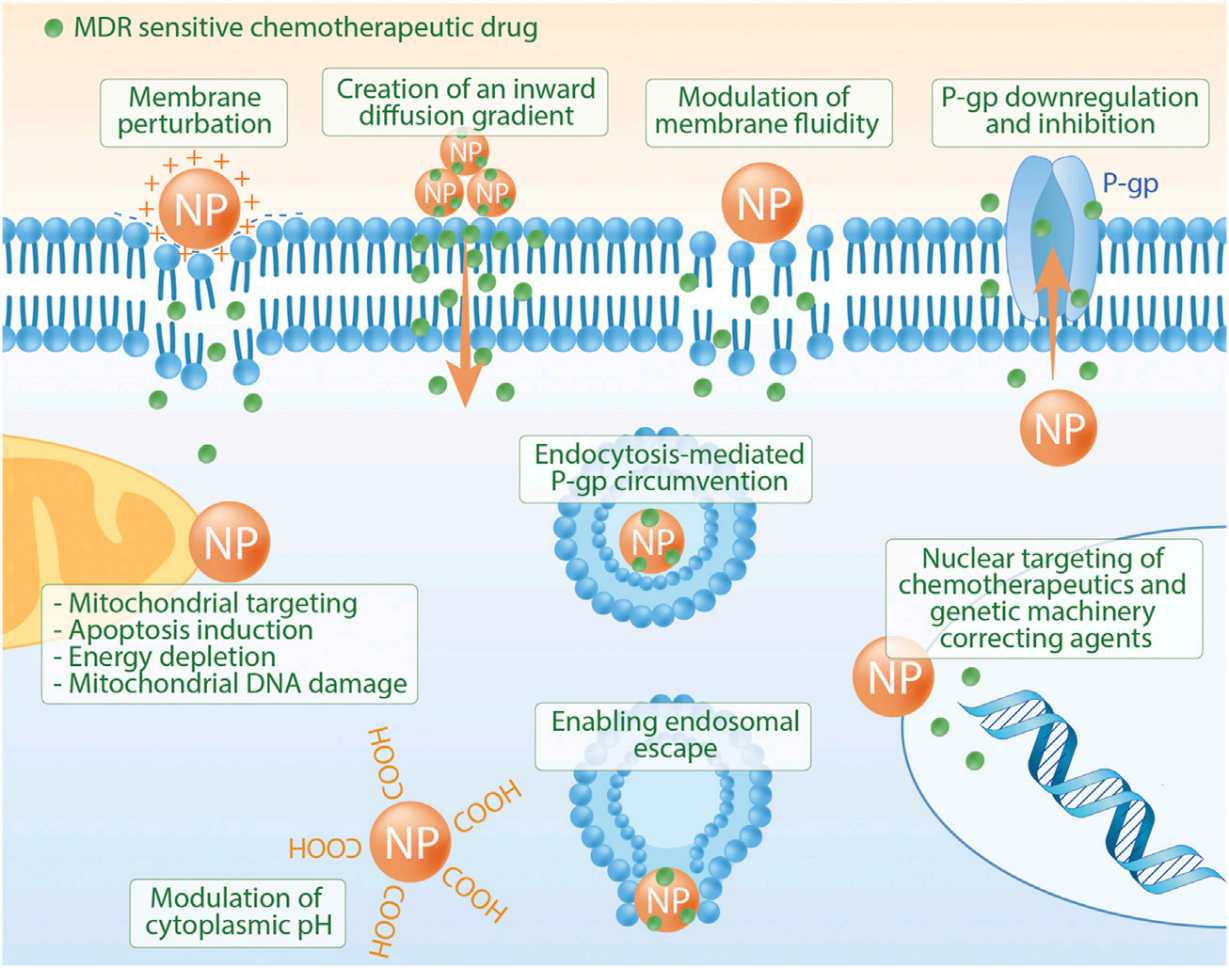
Common nanomedicine strategies to overcome multidrug-resistant tumors. Reprinted from Pharmacological Research, 126, Manu S. Singh, Salma N. Tammam, Maryam A. Shetab Boushehri, Alf Lamprecht, MDR in cancer: Addressing the underlying cellular alterations with the use of nanocarriers, 2-30, Copyright (2017), with permission from Elsevier.

Metal-based NPs such as gold, silver, iron, platinum, and titanium are commonly used for imaging, as drug delivery carriers, and as radiosensitizers in radiation, proton, or photodynamic therapy (Klebowski et al., 2018). Due to their inherent physicochemical properties, metal NPs could overcome MDR via different mechanisms (Sharma et al., 2018), as shown in Figure 2. The endosomal-based cellular uptake mechanism can be considered one of the main advantages of metal-based NPs in overcoming MDR. This is achieved by selectively releasing intracellular drugs after evading the membrane-embedded multidrug efflux pumps (Ayers and Nasti, 2012). Khan *et al.* developed a nanoformulation of superparamagnetic iron oxide nanoparticles (SPION) loaded with curcumin (SP-CUR), an anti-inflammatory and anti-tumorigenic compound. They tested the activity of this nanoformulation in combination with GEM on GEM-resistant pancreatic cells. Efficient delivery of curcumin was achieved using their formulation, and they found that SP-CUR increased the effectiveness of GEM therapy through suppressing two signal transduction pathways that are implemented in MDR: 1) sonic hedgehog (SHH) and 2) oncogenic CXCR4/CXCL12. Following their endosomal escape, SPION particles showed increased cellular internalization where they were observed to be more closely associated with cytosol/mitochondria to prevent lysosomal degradation (Khan et al., 2019). In 2010, Libutti *et al.* used a novel drug delivery system, CYT-6091, surface-modified colloidal gold nanoparticles, to increase tumor levels of rhTNF and reduce its systemic metabolism and toxicity (Libutti et al., 2010). CYT-6091 is constructed by combining rhTNF and thiolyated glycol to the surface of 27 nm gold colloidal particles. CYT9061 was studied at doses between 50 mg/m2 to 600 mg/m2 in a phase I clinical trial conducted with three patients with pancreatic adenocarcinoma. Using the gold colloidal nanoparticles, researchers were able to administer extremely high concentrations of rhTNF compared to when given in isolation: the highest isolated tolerated concentration of pure rhTNF is 1 mg per cycle. After treatment, examination by electron microscope of normal and tumor tissues was carried out and found gold particles isolated within tumor tissues or at anticipated clearance sites (Ladd et al., 2017). These findings suggest that gold-based, colloidal nano-delivery systems can be used to deliver chemotherapy drugs to target tissues. However, subsequent clinical trials remain to be conducted to prove their efficacy and safety.

### Polymeric micelles in PC

Polymeric micelles, including both hydrophobic and hydrophilic moieties (Manzur et al., 2017), are the largest class of nanomaterials being investigated for PC treatment in pre-clinical settings. These nanoparticles contain linear or highly branched and symmetrical polymers, with hydrophilic dendritic extensions and a hydrophobic core. The terminal groups at the dendritic extensions can be adjusted to allow for better solubility depending upon the carrier medium and allow for superior anchorage and permeability into target tissues (Chiba and Twyman, 2017; Ladd et al., 2017). Polymeric micelles are NPs formed by the self-assembly of amphiphilic block copolymers when present in certain solvents with a surfactant concentration above a critical micelle concentration (c.m.c). They are efficient drug delivery systems for cancer treatment with the ability to inhibit P-gp action, alter drug internalization, enable selective drug targeting, and enable subcellular localization. Polymeric micelles can circumvent MDR through a combination of mechanisms, including the EPR effect, endosomal-triggered active internalization, and drug escape (Kapse-Mistry et al., 2014). In our literature review, we found at least seven published studies using polymeric micelles for the delivery of anti-tumor drugs in clinical trials, including Genexol®-PM, NK105, NC-4016, NK012, NC-6004, NK911, and SP1049C (Gong et al., 2012). NK105 is a nanoparticle formulation that incorporates PTX into a “core-shell-type” polymeric micelle. In a phase I trial conducted by Hamaguchi et al. (2007), the pharmacokinetics of NK105, a micelle carrier for PTX, was studied. The study included 19 patients with advanced PC who were given an initial dose of 10 mg/m^2^ of NK105; this dose increased successively. The size of metastatic liver tumors was reduced by 90% in patients given a 150 mg/m^2^ dose of the drug (Hamaguchi et al., 2007).

### Lipid-based nanoformulations in PC

Lipid-based nanoformulations have been extensively studied for the delivery of antitumor agents, including natural products (Kashyap et al., 2019). Liposomes are lipid bilayer systems that can cross the lipid bilayer to selectively target cancer cells based on biomarkers, overly expressed on PC cells (Urey et al., 2017). There are several strategies whereby liposomes can enhance drug bioavailability and efficacy in drug-resistant cancer. These include 1) liposomes modified for controlled and on-demand release; and 2) ligand-targeted liposomes such as immunoliposomes, which facilitate intracellular drug delivery into tumor cells. Liposomes can also directly inhibit P-gp through endocytosis and consequently enhance intracellular drug accumulation (Kapse-Mistry et al., 2014). In 2012, the use of liposomes in PC treatment was explored by Yoshida *et al.* They targeted fucosylated antigens that are highly expressed on the surface of PC cells. They engineered l-fucose-bound liposomes that encapsulated Cy5.5 or cisplatin. *In vitro* studies on CA19-9 expressing PC cells showed that l-fucose-bound liposomes encapsulating either Cy5.5 or cisplatin were effectively delivered and in mouse xenograft models, cisplatin-loaded liposomes were successfully delivered to PC cells and inhibited tumor growth (Yoshida et al., 2012). Furthermore, in the second-line setting of metastatic PDAC following administration of GEM-based regimens, nanoliposomes irinotecan (nal-IRI) was approved by the FDA in combination with 5-FU and leucovorin (5-FU/LV) in 2015. In phase III, the NAPOLI-1 trial, the median progression-free survival (PFS) was 3.1 and 1.5 months (*p* < 0.001) in patients who received nal-IRI + 5-FU/LV and patient’s 5-FU/LV alone, respectively (Wang-Gillam et al., 2016). In the final OS analysis of the NAPOLI-1 trial, the median OS was increased by 2 months (*p* = 0.042) in the nal-IRI + 5-FU/LV group (Wang-Gillam et al., 2016).

### Nanogels in PC

Nanogels can utilize the unique characteristics of tumor microenvironments such as pH and temperature, to release drugs within the cell, resulting in efficient drug delivery (Damaghi et al., 2013). In a study by Damaghi et al., a nanogel-based platform for PC therapy was reported (Soni et al., 2019). They developed a cisplatin-loaded, mAb-coated nanogel for targeted delivery and used it in combination with GEM. *In vitro* results revealed an increase in drug efficacy. Additionally, enhanced drug delivery and synergistic cytotoxic effect were observed after sequential exposure of PC cells to GEM. Together, these studies have all demonstrated the advantage and improved therapeutic outcomes with the use of nanomaterials and nano-drug platforms, particularly and most importantly against MDR in experimental models and clinical trials (Kesharwani et al., 2015; Borsoi et al., 2017; Guo et al., 2018; Kong et al., 2019). Such advantage to the use of nanomaterials is mediated through a wide range of mechanisms, including enhanced cellular uptake, evading endosomal-lysosomal drug breakdown, inhibition of drug efflux, and increasing plasma half-life.

### Key limitations on the clinical translation of nanomedicine in PC

There has been a tremendous effort to understand the structural and functional properties of nanoparticles directed against cancer but their translation to clinical practice has been largely limited. This can primarily be attributed to a poor understanding of the biological barriers and nanomaterial behavior inside the body and cells, as well as the overemphasis and relying on animal models during pre-clinical evaluation, which does not necessarily represent the same disease phenotype in humans (Gonzalez-Valdivieso et al., 2021).

There are several major challenges in the treatment of PC, which need to be overcome to make the use of nanotherapies a success against PCs. These include off-target toxicity, low bioavailability of chemotherapeutic drugs, and undesirable pharmacokinetics. One way to address these obstacles is through the use of nanotechnology as an effective vehicle for chemotherapeutic drugs. Currently, only 16 nano-based cancer drugs are approved by FDA and around 75 nanoformulations are being investigated in clinical trials (He et al., 2019). It is extremely important to narrow the gap between preclinical toxicity studies and toxicity studies in patients, as nanomedicines have been shown to exert additional unintended and often toxic effects on normal cellular function. Moreover, there has been a lack of convincing data on the process of excretion of nanomedicines from the human system, as most data is available from animal disease models. Nanomedicines can pose safety issues at different levels (apart from the intrinsic toxicity of the API itself). Furthermore, the biodistribution of nanoparticles changes unpredictably resulting in uptake and accumulation in certain organs, which may result in target off-target effects and local overexposure. Indeed, some nanoparticles have a known tendency to accumulate in lymphoid organs and kidneys (for some polymer-bound drugs) (Metselaar and Lammers, 2020).

Furthermore, there remains a general lack of understanding on the cost-effectiveness, manufacturing, and scaling up, as well as regulation with regard to using nanomedicines for cancer (van der Meel et al., 2017). As the science behind the structural-functional relationship provides clarity on the interaction of nanomedicines *in vivo*, the regulatory challenges must be addressed simultaneously to bring these potentially game-changing therapeutics to the frontline against fighting pancreatic cancer.

## Conclusion and future directions

Numerous noncarriers have been developed and investigated for the treatment of PC to overcome the problem of MDR seen with chemotherapy and other therapeutic options, however, with limited success Even though nano-based carriers show great promise in treating various cancers, they have several limitations, including potential toxicity, difficult scalability, and low loading efficiency that could be responsible for their low success rate reaching clinical settings. Notably, the albumin-based nanocarrier was the most successful in clinical studies for PC. This is mainly because albumin nanoparticles were successful in encapsulating widely used chemotherapeutics that are less soluble/insoluble in water. Additionally, albumin is highly biocompatibility and biodegradability, making it an attractive material for drug delivery applications. The development of future therapies for cancer and nano-based therapeutics should not be limited by designing nanocarriers only for passive targeting of cancerous cells. Internalization of chemotherapeutic agents into tumor cells can be further improved via the utilization of an active targeting approach to enhance drug delivery efficacy. Additionally, research into novel biomarkers to enable active targeting will empower delivery strategies of nanocarriers to combat cancer resistance.

While we have certainly made huge progress in understanding the drug resistance mechanisms in PC and the signaling pathways responsible for PC cell metastases, investigations on the use of nanomedicine in this field lag behind. Currently, the majority of work in the field of nanomedicine is largely focused on increasing drug stability, accumulation, and targeting, which is yet of critical importance, particularly in nucleoside transporter (e.g., ENT1 and CNT1)-mediated drug resistance against GEM (Hung et al., 2015; Poon et al., 2015). Future studies utilizing nanotechnology against MDR pancreatic cancers should integrate multiple modalities and exploit the rapidly accumulating mechanistic knowledge in this cancer model (e.g., targeting PCSCs, dual delivery of potential drug modalities, etc.). In addition, utilizing the endogenous properties of the TME to trigger the release of cancer therapeutics from nanocarriers should be considered during the delivery system design. Such a design will add another dimension of controlled release that can impact clinical efficacy, where adverse effects can be minimized while retaining therapeutic benefits.

The main goal of PC treatment is to enhance the efficiency of drug delivery and minimize drug resistance. Despite the tremendous effort in making novel nanocarriers in pre-clinical settings, the development of clinical translation to the bedside remains laborious. Extrapolation of scientific findings from animals to humans is extremely challenging, mainly due to differences in physiology and anatomy between species, making direct extrapolation unreliable. Furthermore, unlike the experimental settings in clinical studies, animals are designed with syngeneic backgrounds, and disease models are designed to produce as homogenous a population as possible. On the other hand, heterogeneity is the basis of ineffectiveness in clinical trials. Moreover, individual variability in lifestyle and disease progression plays key roles in the overall efficacy, unlike the well-controlled animal experiments.

The promise of nanomedicine will be realized by moving away from designing a targeting strategy against a single target to including targeting approaches that address multiple signaling mechanisms and molecular targets, considering the complexity of both the human physiology and the tumor microenvironment, including the development of MDR mechanisms.
